# Light-Controlled Soft Switches for Optical Logic Gate Operations

**DOI:** 10.3390/s25072051

**Published:** 2025-03-25

**Authors:** Chuang Wang, Hao Wu, Quanwang Niu, Xiaohong Yan, Xiangfu Wang

**Affiliations:** 1 College of Electronic and Optical Engineering & College of Flexible Electronics (Future Technology), Nanjing University of Posts and Telecommunications, Nanjing 210023, China; 1222025018@njupt.edu.cn (C.W.); wh826214336@163.com (H.W.);; 2 Key Laboratory of Radio Frequency and Micro-Nano Electronics of Jiangsu Province, Nanjing 210023, China

**Keywords:** light-driven soft actuator, liquid crystal elastomers, carbon nanotubes, logic gate

## Abstract

While liquid crystal elastomers (LCEs) show promise for diverse soft actuators due to their strong stimulus responsiveness, limited investigation into their light perception and processing restricts their wider use in intelligent systems. This study employs a hollow double-layer structure to design light-controlled logic soft switches based on LCEs. The design realizes digital logic circuits including AND gates, OR gates, and NOT gates, as well as an optical switch array capable of converting light signals into visualized digital signals. These light-controlled soft switches exhibit strong photothermal responsiveness (~12 s), high programmability, and excellent cyclic stability (>500 times). This research provides a new perspective on light-controlled logic soft switches and their applications in logic circuits.

## 1. Introduction

Logic switches are essential components in flexible electronic devices, playing a significant role in information processing and logical operations [1,2,3]. However, traditional logic switches are primarily constructed from semiconductor devices, making them susceptible to external environmental factors such as humidity, electromagnetic interference, and high voltage, which leads to poor adaptability in various conditions [4,5]. To tackle this issue, researchers have actively explored and developed logic switches based on principles such as light [6,7,8,9], heat [10,11], mechanics [12,13,14], and chemistry [15]. Among these, light-controlled switches offer the advantage of remote and precise control through the use of light while also minimizing the risk of mechanical wear and physical damage. This enhancement leads to improved stability and extended lifespan of the switches.

In recent years, researchers have developed a variety of light-controlled logic switches. Wu et al. [6] demonstrated a photochromic bisthiazolethene system, which absorbs across the entire UV-visible spectrum. Molecules embedded in polymer films can function as AND or NOT gates in response to different time intervals. Fei et al. [7] introduced azobenzene light-responsive molecules in three sets of thermochromic systems, utilizing the photoisomerization properties of azo compounds to achieve light-controlled thermochromic functionality. Tholen et al. [8] proposed a method for embedding combinational logic circuits within liquid crystal elastomer films. These films are designed as integrated switches and use liquid metal conductive traces for circuit integration, enabling combinational logic operations. However, light-controlled logic switches are currently limited primarily to molecular-scale photochromism. Photochromic molecules, upon repeated exposure to light cycles, can undergo chemical decomposition or isomerization, leading to a decline in performance and consequently impacting the efficiency of the device’s overall logical operation. Additionally, these switches have limited scalability and face challenges in integration into practical application systems. When considering light sources, ultraviolet light presents issues such as low biosafety, poor penetration capability, and high energy consumption.

In this work, we designed a light-controlled logic soft switch based on liquid crystal elastomers (LCEs), featuring a hollow double-layer structure composed of an actuation layer and a sensing layer. Compared to traditional double-layer structures [16,17,18], the hollow design provides a more sensitive deformation response, as the internal cavity allows for greater deformation amplitude under the same external forces or conditions. This structure enables enhanced flexibility in adjusting the materials and geometries of both the inner and outer layers, allowing for precise control of the soft actuator. This actuation layer consists of carbon nanotube-doped LCEs and copper tape, while the sensing layer is made of copper foil. Recently, photothermal components such as carbon nanotubes (CNTs) and graphene oxide (GO) have been incorporated into LCEs to enhance actuation performance, owing to their exceptional photothermal conversion efficiency. These nanomaterials can efficiently absorb light energy and convert it into thermal energy, thereby rapidly and precisely triggering the deformation of LCEs, significantly augmenting actuation capabilities [19,20,21,22,23]. This photothermal effect accelerates the temperature rise of the LCEs, further enhancing their deformation speed and amplitude, thereby improving the performance of the soft switch. By controlling the intensity [24,25,26], wavelength [27,28,29], and exposure time of the light [30,31,32], we can modulate the degree of deformation, thereby enabling the switching of the light-switch states. Consequently, the LCE-based soft switch effectively processes optical information through the conversion of light, heat, and mechanical energy. The soft switch can be configured to form optical logic gates, facilitating logical operations such as AND, OR, and NOT. Moreover, arrays of soft switches can be integrated with electronic components to enable the acquisition, processing, and display of optical information.

## 2. Materials and Methods

The synthesis of CNT-LCE composite material requires the following components: diacrylate liquid crystal mesogen RM257 (1,4-bis-[4-(3-acryloyloxypropyloxy)benzoyloxy]-2methyl-benzene, 97%, Aladdin Biochemical Technology Co., Ltb., Shanghai, China), chain extender EDDET (2,2′(ethylenedioxy) diethanethiol, 97%, Aladdin Biochemical Technology Co., Ltb., Shanghai, China), cross-linker PETMP (pentaerythritol tetrakis (3-mercaptopropionate), 95%, Aladdin Biochemical Technology Co., Ltb., Shanghai, China), catalyst DPA (Dipropylamine, 99%, Aladdin Biochemical Technology Co., Ltb., Shanghai, China), a photoinitiator (2-Hydroxy-4′-(2-hydroxyethoxy)-2-methylpropiophenone, 98%, Aladdin Biochemical Technology Co., Ltb., Shanghai, China), multiwalled carbon nanotubes (MWCNT, 90%, Aladdin Biochemical Technology Co., Ltb., Shanghai, China), toluene (Aladdin Biochemical Technology Co., Ltb., Shanghai, China), copper tape, and copper foil.

**Figure 1:**
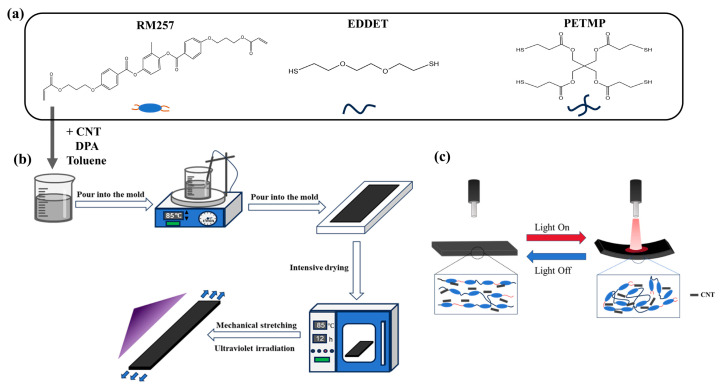
(**a**) Main components and molecular structure of the LCE. (**b**) Preparation process of the CNT-LCE composite material. (**c**) Schematic illustration of the reversible shape-bending deformation of CNT-LCE under near-infrared light irradiation.

In this experiment, for the synthesis of LCEs, the molar ratio of the components was RM257: EDDET: PETMP = 55%: 42%: 3%. The preparation process is illustrated in Figure 1a,b. First, 2 g of RM257 was mixed with 0.7 g of toluene and stirred magnetically at 85 °C for 10 min to ensure complete dissolution of RM257 in the toluene. Next, CNTs were added to the mixed solution, and the mixture was stirred again for 10 min to achieve uniform dispersion of the CNTs. Subsequently, 0.474 g of EDDET, 0.098 g of PETMP, and 0.012 g of photoinitiator were added, and the mixture was stirred at 85 °C until all components were fully dissolved. Afterward, 0.288 g of DPA was added as a catalyst. Once thoroughly mixed and degassed, the solution was carefully poured into pre-prepared molds. The molds were placed in a sealed container overnight to ensure complete reaction. The mixture was then removed from the molds and placed in a vacuum drying oven at 85 °C for 12 h. After the LCE was dried, it was uniaxially stretched to 100% strain. Finally, the material was exposed to 365 nm UV light for 1 h. The resulting CNT-LCE film was used for subsequent experiments.

In uniaxially aligned LCEs, uniform heating to the liquid crystal-isotropic phase transition temperature, followed by cooling back to the liquid crystal phase, induces predictable contraction or extension deformation along the liquid crystal alignment direction. However, when utilizing photothermal actuation, the deformation behavior of the strip actuators exhibits significant variations due to the material’s light absorption characteristics and the spatial selectivity of the illumination. Specifically, the following deformation modes are observed: (1) reversible bending towards the light source under illumination, (2) reversible bending away from the light source under illumination, and (3) reversible contraction and extension under applied load. These diverse deformation behaviors underscore the advantages of photothermal actuation in precisely controlling the actuation performance of LCEs. Under stimulation by near-infrared light, the exceptional photothermal effect of CNTs allows them to absorb light energy and rapidly heat up, resulting in a localized temperature increase in the CNT-LCE composite. The LCE is highly sensitive to temperature; as the temperature rises, the liquid crystal molecules within the LCE transition from an ordered arrangement (liquid crystal phase) to a disordered arrangement (isotropic phase) [33,34,35]. As shown in Figure 1c, this phase transition leads to a macroscopic deformation of the LCE, causing the CNT-LCE film to bend in the direction of the near-infrared light irradiation. When the light source is turned off, the CNTs cease to absorb light energy, and the temperature begins to decrease. As the material cools, the liquid crystal molecules return to their ordered arrangement, allowing the LCE to revert to its initial liquid crystal state, and the material’s shape reverts to its original form. Through cycles of illumination and cooling, the material can undergo repeated deformation and recovery.

**Figure 2:**
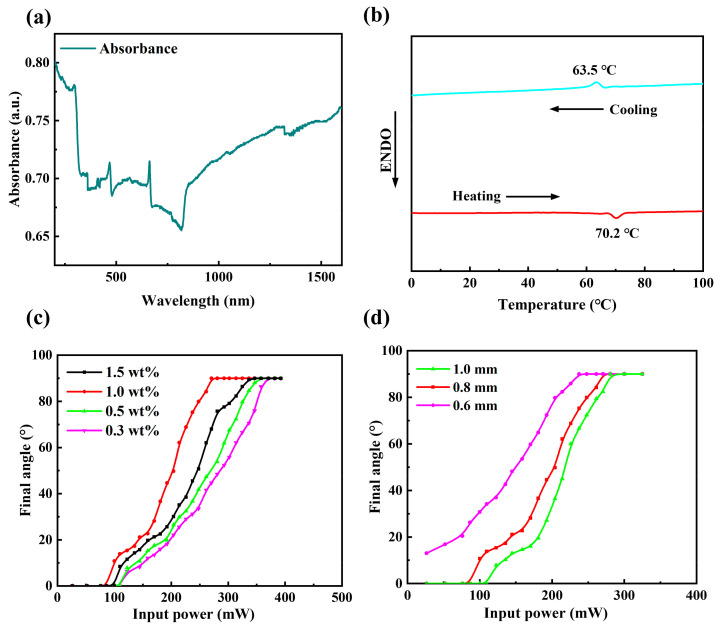
(**a**) UV-visible–near-infrared absorption spectrum of the CNT-LCE film. (**b**) Data plots of DSC measurement upon heating and cooling scans of the 1 wt% CNT-LCE. (**c**) Relationship between input power and the final bending angle of CNT-LCE with varying concentrations of doped CNT. (**d**) Relationship between input power and the final bending angle of CNT-LCE at different film thicknesses.

The molds for curing the LCE were fabricated using a digital light processing 3D printer (ShapeCure, Laisai Intelligence Technology Co., Ltd., Suzhou, China). The absorbance of the CNT-LCE composite was characterized using a UV-visible–near-infrared spectrophotometer (UV3600, Shimadzu Research Laboratory Co., Ltd., Shanghai, China), as shown in Figure 2a. As depicted in the figure, CNT-LCE exhibits significant light absorption properties across both infrared and ultraviolet spectral ranges. Within the infrared spectrum, common laser sources are primarily concentrated at wavelengths of 808 nm, 980 nm, and 1545 nm. Comparative analysis reveals that the laser absorption rate of CNT-LCE at 1545 nm is markedly higher than that at 808 nm and 980 nm. Given the potential for ultraviolet radiation to induce damage in biological tissues, this study opted for a 1545 nm laser (semiconductor infrared laser, MW-GX-1545, Changchun Laser Technology Co., Ltd., Changchun, China) as the experimental light source to ensure both experimental safety and biocompatibility. Infrared thermal images of the CNT-LCE under near-infrared irradiation were captured using a handheld infrared thermal imager (HM-TPK20-3A QF/W, Hikvision Digital Technology Co., Ltd., Hangzhou, China) to determine the localized maximum temperature. All photographs of the physical samples were taken at room temperature using a smartphone (Xiaomi 13 Ultra, Xiaomi Technology Co., Ltd., Beijing, China).

To investigate the impact of CNT doping on the thermodynamic properties of LCEs, a different scanning calorimetry (DSC) analysis was performed on CNT-LCE composites with a doping concentration of 1 wt%. As illustrated in Figure 2b, a distinct endothermic peak was observed during the heating process, with a peak temperature of 70.2 °C, corresponding to the transition temperature from the liquid crystalline phase to the isotropic phase. Conversely, during the cooling process, an exothermic peak appeared at 86.5 °C, indicative of the transition from the isotropic phase back to the liquid crystalline phase. The DSC results unequivocally demonstrate that the CNT-LCE composite exhibits characteristics of a liquid crystalline phase, thereby confirming its thermotropic liquid crystalline behavior.

## 3. Results and Discussion

### 3.1. CNT-LCE Actuation Performance Test

To accurately evaluate the photoactuation performance of the material, a vertical incidence configuration was employed, wherein the laser source was positioned directly above the material at a 90° angle to the horizontal plane. The irradiation distance from the laser source to the sample was maintained at 3 cm, and the irradiation area was controlled to 1.368$\;{\mathrm{cm}}^{2}$. When fabricating thelight-controlled logic soft switch, the actuation performance of the CNT-LCE in the actuation layer is crucial for the responsiveness of the soft switch. First, we investigated the effect of the CNT doping concentration on the bending performance of the actuation layer. In this experiment, we prepared four types of LCE doped with different concentrations of CNTs—0.3 wt%, 0.5 wt%, 1 wt%, and 1.5 wt%—with all samples maintaining a consistent thickness of 1 mm. As shown in Figure 2b, all four CNT-LCEs exhibited good bending performance, achieving a bending angle of 90° under specific near-infrared light power, in alignment with the angle of the incident light. However, there were significant differences in the relationship between input power and actuation angle among the four materials. For CNT-LCEs with doping concentrations below 1 wt%, the actuation angle increased with higher doping concentrations at a fixed laser power. The lower concentrations of 0.3 wt% and 0.5 wt% showed weaker photothermal response due to insufficient dispersion and less significant photothermal effects of the CNTs, resulting in smaller actuation angles. In contrast, the CNT-LCE with a doping concentration of 1.5 wt% exhibited a larger actuation angle at a fixed laser power compared to the 0.3 wt% and 0.5 wt% samples due to the enhanced photothermal responsiveness associated with higher concentrations of CNTs. However, compared to the 1 wt% CNT-LCE, the increase in CNT concentration also resulted in greater material rigidity, which restricted the free movement of liquid crystal molecules, thereby affecting the deformation performance of the actuation layer. This led to the 1 wt% CNT-LCE exhibiting a greater actuation angle. The experimental results indicate that the doping concentration of CNTs significantly impacts the actuation performance of the actuation layer, with 1 wt% being the optimal concentration, as it achieves substantial deformation at lower power levels, demonstrating superior actuation performance.

The thickness of the CNT-LCE also significantly affects the bending performance of the actuation layer. As shown in Figure 2c, at the same laser power, the deformation angle of the 0.6 mm CNT-LCE is greater than that of the 1 mm and 0.8 mm CNT-LCEs, demonstrating optimal actuation performance. This is because thinner CNT-LCEs can respond more rapidly to external photothermal stimuli. This quick response allows the material to reach the desired temperature more swiftly under the same laser power, resulting in a larger deformation angle. In contrast, the thicker 1 mm and 0.8 mm CNT-LCEs, while capable of absorbing more heat due to their larger volumes, exhibit a greater internal temperature gradient. The heat requires more time to transfer from the surface to the interior, leading to the need for higher power levels to overcome this thermal conduction bottleneck and trigger the material’s deformation.

**Figure 3:**
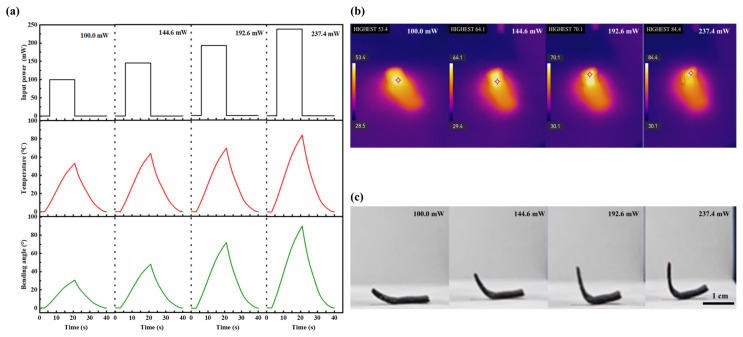
(**a**) Variation of temperature and bending angle of the CNT-LCE actuation layer over time under 100 mW, 144 mW, 192.6 mW, and 237.0 mW of 1545 nm near-infrared light power. (**b**) Infrared thermal image and (**c**) photograph of CNT-LCE under near-infrared light irradiation.

Investigating the effects of the CNT doping concentration and thickness on the bending performance of the CNT-LCE actuation layer evidences that the CNT-LCE with a doping concentration of 1 wt% and a thickness of 0.6 mm exhibits superior actuation bending performance. Next, we analyze the response characteristics of this CNT-LCE actuation layer under different input light power levels, specifically focusing on the variations in input power, temperature, and bending angle. As shown in Figure 3, when subjected to near-infrared light at power levels of 100.0 mW, 144.6 mW, 192.6 mW, and 237 mW at a wavelength of 1545 nm, the surface temperature of the CNT-LCE was uniformly heated to 53.4 °C, 64.1 °C, 70.1 °C, and 84.4 °C, respectively. It is evident that higher input power results in a faster rate of temperature increase and a higher peak temperature, indicating a positive correlation between input light power and temperature. Under the near-infrared light at 100.0 mW, 144.6 mW, 192.6 mW, and 237 mW, the maximum bending angles achieved by the CNT-LCE were 30.7°, 48.1°, 72.4°, and 90°, respectively. The trend in bending angle closely follows the trend in temperature change, with both gradually recovering after reaching their peak values, further confirming the positive correlation between input light power and bending angle. By adjusting the input power, it is possible to precisely control the temperature and bending angle of the CNT-LCE, enabling different driving effects.

### 3.2. Optical Switch Performance Test

**Figure 4:**
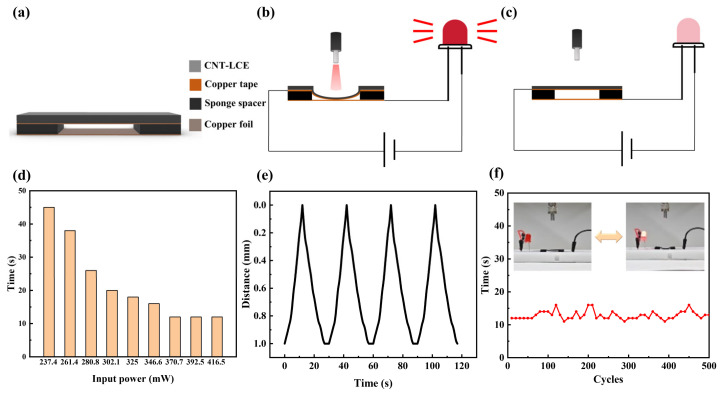
(**a**) Schematic diagram of the soft optical switch structure. (**b**,**c**) Illustrations of the working principle of the soft optical switch. (**d**) Response time of the soft switch under different light power levels. (**e**) Periodic changes in the distance between the actuation layer and the sensing layer under 379.7 mW laser illumination over time. (**f**) Repetitive testing of the soft optical switch.

The switch is the fundamental unit for constructing any combinatorial logic operation. In this study, the structure of the light-controlled logic soft switch based on LCEs comprises an actuation layer and a sensing layer. As shown in Figure 4a, the actuation layer consists of an LCE doped with CNT and copper tape. The LCE is selected with an optimal doping concentration of 1 wt% and a thickness of 0.6 mm, while the sensing layer is made from copper foil with a thickness of 0.05 mm. In the structural design of the device, two sponge spacer layers serve as a critical supporting component, effectively separating the actuation layer (CNT-LCE) and the sensing layer (copper foil). This spacer layer not only provides the necessary space for the deformation of the actuation layer, ensuring its free bending under illumination, but also maintains the overall mechanical stability of the structure. Furthermore, the sponge spacer significantly reduces direct contact between the two material layers, effectively preventing potential issues such as friction, wear, or adhesion, thereby extending the device’s lifespan. The electrical connection of the device is achieved through contact between the middle sections of the upper and lower layers. When the photoactuation layer undergoes deformation, the upper and lower layers come into contact, forming a closed circuit and realizing the switching function. The operating principle of the soft optical switches is illustrated in Figure 4b,c. The actuation layer is connected to the positive terminal of the power supply, while the sensing layer is connected to a light-emitting diode (LED) and the negative terminal of the power supply. When 1545 nm near-infrared light illuminates the optical switch, the actuation layer receives the light signal. The CNT-LCE absorbs the light energy and rapidly heats up, leading to a localized temperature increase that causes deformation and downward bending. Under continuous illumination, the actuator bends down to contact the sensing layer, triggering the LED to emit light, indicating that the circuit is closed. When the light source is turned off, the CNT-LCE stops absorbing light energy, and the surface temperature of the actuation layer gradually decreases due to the influence of the surrounding environment. As a result, the actuation layer begins to return to its original position, and the light emission from the diode extinguishes, indicating that the circuit is open.

**Figure 5:**
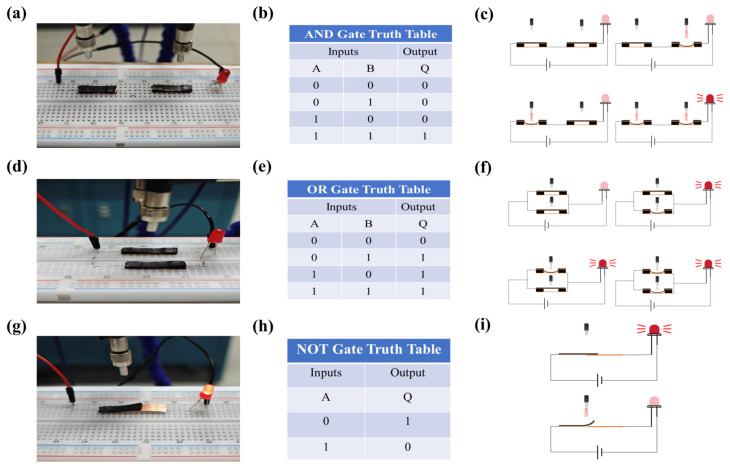
(**a**) Photograph of the soft optical switch configured as an AND gate. (**b**) Truth table for the AND gate. (**c**) Illustrations of the working principle of the AND gate based on the LCE optical switch. (**d**) Photograph of the soft optical switch configured as an OR gate. (**e**) Truth table for the OR gate. (**f**) Illustrations of the working principle of the OR gate based on the LCE optical switch. (**g**) Photograph of the soft optical switch configured as a NOT gate. (**h**) Truth table for the NOT gate. (**i**) Illustrations of the working principle of the NOT gate based on the LCE optical switch.

To ensure that the soft optical switch achieves a sufficiently fast driving speed, we selected a 1 mm thick sponge as the spacer layer in the hollow double-layer structure. Additionally, an appropriate laser power must be chosen as the stimulus for the soft optical switch. As shown in Figure 4d, when the laser power is set at 237.4 mW, the response time of the soft optical switch is 45 s. As the laser power increases, the response time gradually decreases. When the laser power reaches or exceeds 370.7 mW, the response time stabilizes at 12 s. Therefore, we selected 370.7 mW of 1545 nm laser light as the stimulus for subsequent experiments. As illustrated in Figure 4e, when illuminated, the distance between the actuation layer and the sensing layer gradually decreases, reaching 0 mm after 12 s, at which point the diode lights up. Upon removing the light source, the distance between the two layers gradually increases, returning to 1 mm after 15 s. The repeatability of the soft optical switch is also critical. Figure 5f shows the results of 500 switching tests conducted on the soft optical switch, demonstrating that the response time consistently remains around 12 s, including excellent repeatability performance.

### 3.3. Application

The light-controlled logic soft switch based on LCE exhibit light-responsive properties that enable shape deformation, thereby altering the state of an electrical circuit. These characteristics allow for the construction of fundamental logic gates, such as AND, OR, and NOT gates, for the execution of various digital logic operations. As illustrated in Figure 5a, two soft optical switches are connected in series to form an AND gate, connected to an LED, with the LED on/off state indicating whether the logic operation is satisfied. Figure 5b presents the truth table for the AND gate. In Figure 5c, the LED illuminates only when both switches are activated (both inputs are 1), reflecting the AND gate’s output of 1 exclusively under this condition. These illustrations confirm that the AND gate formed by the light-controlled logic soft switches based on LCE adheres to logical operations—the output is 1 only when both inputs are 1. Figure 5d depicts the configuration of two soft switches in parallel to create an OR gate, also connected to an LED. The diode’s illumination reflects the logical operation of the OR gate. The truth table for the OR gate is presented in Figure 5e. As seen in Figure 5f, the LED only remains unlit when both switches are deactivated. The LED illuminates whenever one or both switches are activated. These figures demonstrate that the OR gate, constructed from light-controlled logic soft switches based on LCE, fulfills the logical operation where the output is 1 if at least one input is 1. In the design of the NOT gate, as depicted in Figure 5g, the actuation layer and sensing layer are stacked, with the actuation layer on top and the sensing layer on the bottom. Figure 5h presents the truth table for the NOT gate. As shown in Figure 5i, when the switch is not activated, the circuit is closed, and the LED illuminates. Conversely, when the switch is activated, the circuit opens, and the LED is extinguished. This aligns with the NOT gate’s logic: the output is 1 when the input is 0, and the output is 0 when the input is 1.

**Figure 6:**
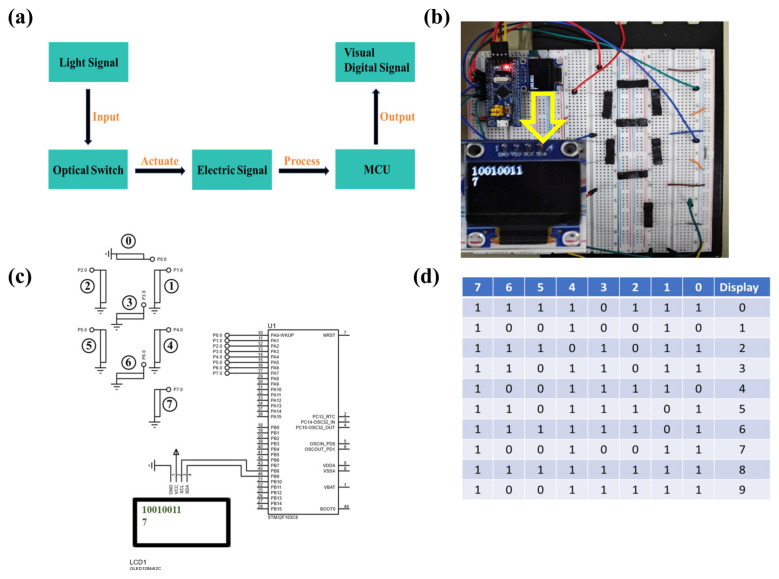
(**a**) Workflow diagram of the soft optical switch array. (**b**) Physical diagram of the soft optical switch array and microcontroller. (**c**) Schematic diagram of the soft optical switch array and microcontroller. (**d**) Encoding table for the light-signal display of the soft optical switch array.

Light-controlled logic soft switch arrays can be configured in combination with electronic components, facilitating the collection, processing, and display of optical information. Figure 6a illustrates the workflow of the light-switch array, where external optical signals serve as input. Upon optical stimulation, the soft switches undergo deformation, converting external optical signals into electrical signals. These electrical signals are then transmitted to a microcontroller (STM32F103C8) for processing, with the processed results visualized as digital signals on an OLED display. Figure 6b presents a physical depiction of the fabricated optically controlled logic soft switch array. As illustrated, the soft switch array and the microcontroller are precisely positioned and affixed to a circuit board, with reliable connections established between the soft switch array and the microcontroller via multiple data lines. The soft switch array is arranged in a seven-segment display configuration, facilitating the input of digital signals. The microcontroller, serving as the central control unit, is responsible for receiving and processing electrical signals from the soft switch array and subsequently driving an OLED display screen to present the results (The yellow arrow points to the number displayed on the current screen). Figure 6c details the arrangement, with the soft switches organized in an array resembling a seven-segment display. Eight soft switches are numbered; positions 0~6 represent the switch locations, while position 7 serves as the confirmation button. For instance, to transmit the signal for the 7 digit, light stimulation is applied to switches numbered 0, 1, and 4, followed by stimulation of switch 7, indicating the completion of optical signal input. Upon processing of the signals, the microcontroller displays two lines of information on the OLED screen: the first line indicates the response status of the soft switches (1 for light stimulation and 0 for no stimulation), while the second line shows the initially transmitted signal (the digit 7). Figure 6d provides a coding table for the optical signals displayed by the soft switch array, illustrating how digit signals from 0 to 9 can be transmitted and displayed through optical stimulation. These results demonstrate that the light-switch array system efficiently converts optical signals into digital signals for display, highlighting its potential applications in intelligent detection and display systems. The findings indicate that the light-switch array is capable not only of efficiently collecting and processing optical information but also of enabling intelligent processing and display via the microcontroller. In the current design, the logic switches based on LCEs exhibit relatively large dimensions, which may limit their application in miniaturized systems such as wearable electronic devices. Despite the current size constraints, LCE-based logic switches still hold potential application value in fields such as flexible robotics, sensors, and actuators.

## 4. Conclusions

In this work, we designed a light-controlled logic soft switch based on LCE using a hollow double-layer structure, enabling the implementation of AND, OR, and NOT digital logic circuits, as well as a switch array capable of converting optical signals into visual digital signals. The LCE soft optical switch can achieve precise logic operations under optical stimulation, successfully converting input signals into corresponding output signals. The realization of the AND, OR, and NOT digital logic circuits validates the application potential of LCE soft optical switches in logic circuits. Furthermore, the switch array system successfully displayed the corresponding digital signals on the screen through the input, conversion, and processing of optical signals. Thus, this study provides new insights into light-controlled logic soft switches and their applications in logic circuits, promoting advancements in the field of smart flexible devices.

## Data Availability

Data are contained within the article.
